# The Deeper the Love, the Deeper the Hate

**DOI:** 10.3389/fpsyg.2017.01940

**Published:** 2017-12-07

**Authors:** Wang Jin, Yanhui Xiang, Mo Lei

**Affiliations:** ^1^Center for Studies of Psychological Application, South China Normal University, Guangzhou, China; ^2^Guangdong Key Laboratory of Mental Health and Cognitive Science, South China Normal University, Guangzhou, China; ^3^School of Psychology, South China Normal University, Guangzhou, China; ^4^Cognition and Human Behavior Key Laboratory of Hunan, Department of Psychology, Hunan Normal University, Changsha, China

**Keywords:** romantic love, romantic hate, similarity, connection, emotional reactions

## Abstract

Love and hate are basic human affects. Previous research has focused on the classification, functions, and other aspects of these two affects. However, few studies have been conducted on the relationship between love and hate. The present study investigated whether similarity within romantic partners was associated with greater feelings of love in the absence of betrayal, and greater hate induced in the presence of betrayal by using vignettes to induce love and hate in a sample of 59 young adults. The results showed that people who shared similar values and interests with the target persons were more likely to experience stronger love. Additionally, stronger feelings of love were associated with greater hate after the relationship was broken, suggesting a link between romantic love and hate. Our study revealed a complex picture of love and hate. People have different emotional reactions toward different target persons in the context of romantic love and hate. If one loves someone deeply and sometimes hates that person, the feeling of love may still be dominant in the context of betrayal. However, if one does not love that person, hate will be a much stronger feeling than love.

## Introduction

Love and hate are important human affects that are of long-standing interest in psychology. Increasingly, empirical research has been carried out on the relationship between love and hate. However, traditional psychological theories have mainly focused on love, especially romantic love. These include Sternberg’s (1986) triangular theory of love and the three-stage model of love (Fisher, 1989; Fisher et al., 2006). Love has been defined as an action (Swensen, 1972), attitude (Rubin, 1970), experience (Skolnick, 1978), and even as a prototypical emotion (Fehr and Russell, 1991; Post, 2002; Sober, 2002; Wyschogrod, 2002). Collectively, these definitions suggest that love is a multi-faced phenomenon (Ekman, 1972; Izard, 1977; Tomkins, 1984). Hate, within the context of a romantic relationship, arises mainly from a relational betrayal. Researchers have proposed a concept related to romantic hate, romantic jealousy, which describes the negative attitudes, anger, and fear associated with having a relationship partner (Yoshimura, 2004).

Love and hate are related to each other in a complex manner; the methodological approaches used by previous researchers have limited effectiveness in exploring the intricate relationship between love and hate. In addition, there has been little research on the psychological mechanisms that could explain the interrelations between love and hate. Therefore, our study investigates how these two affects are related. To pursue such a research objective, one must consider how best to induce varying levels of feelings of love.

Previous studies have found that attraction is a crucial condition for the development of romantic love (Cutler et al., 1998; Braxton-Davis, 2010; Miller and Maner, 2010). Similarity, rather than complementarity, plays a key role in attraction (Berscheid and Reis, 1998; Luo and Klohnen, 2005; Hudson et al., 2014). Many aspects of similarity have been studied in relation to attraction. In the current study, we focused on similarity in ideologies. That is, persons with similar ideologies (defined here in terms of values and interests) tend to form longer lasting and more harmonious relationships (Buunk and Bosman, 1986; Lemay and Clark, 2008). Ideological similarity also implies commonalities in behaviors which further contribute to mutual attraction in the context of romantic love (Schafer and Keith, 1990). From this perspective, similarity may be a key factor that influences the degree of love. In addition, researchers found that differences in excellence levels, such as those relating to ability and achievement, between partners would also be an important factor influencing romantic relationships (Conroy-Beam et al., 2016).

In the present study, we manipulated the level of similarity and the level of excellence to induce different levels of love. That is, we concurrently varied the levels of similarity and excellence of different targets. We explored whether participants felt stronger love for a target who was more similar to themselves when the targets and participants were of the same level of excellence. Additionally, we were also interested in whether participants have different emotional reactions toward different target persons in the context of romantic love and hate.

We examined two research questions in the current research. First, would there be greater feelings of love between two persons if they were more similar to each other? Second, under certain conditions, does a person’s love generate a corresponding level of hate when negative events occurred to his or her romantic partner?

In this study, we implemented a paradigm similar to what has been used in previous research (Takahashi et al., 2009), and adapted the scenario method to induce love and hate. The characters in the scenario included one protagonist and three targets. Participants read the scenario and imagined that they were the protagonist and were in a romantic relationship with one of the target. We induced different levels of love by manipulating the degree of similarity (e.g., values and interests) and excellence (e.g., ability and achievements) between the protagonist and target persons in the vignettes. We also induced hate using vignettes that showed target persons betraying the protagonist, such as going on dates or having affairs with people of the opposite-sex. We hypothesized that greater similarity between a participant (protagonist) and a target would be associated with greater feelings of love, and that when negative events occur with the protagonist’s romantic partner, the target would be associated with greater feelings of hate.

## Materials and Methods

### Participants

Sixty volunteers, recruited from different colleges, participated in the experiment. One participant had misunderstood the instructions and was thus excluded from the analyses. As a result, the final studied sample consists of 59 participants (30 men, 29 women, age *M* = 20.2 years, *SD* = 1.5). None of the participants reported any previous diagnoses of psychiatric or neurological illnesses. Roughly 18% of the participants said they were looking for a relationship, 33% were in a relationship, 24% had experienced a break-up, and the remaining 25% had not been in any relationships. The study was approved by the Ethics Committee of the School of Psychology at South China Normal University. Each participant had provided written informed consent prior to participating in the experiment. They were also given small tokens of appreciation for their participation.

### Materials

The vignettes used in the present experimental paradigm were adapted from a previous study that investigated the neural correlates of envy and schadenfreude (Takahashi et al., 2009). The vignettes were modified to fit the present romantic love context, according to the previous definitions of love (Hatfield and Sprecher, 1986; Schafer and Keith, 1990). The people in the vignettes included one protagonist and three targets (i.e., targets A, B, and C) corresponding to three manipulated conditions (see Supplementary Material). Participants were asked to study and understand the vignettes thoroughly and to imagine themselves as the protagonist in the vignettes. Target A was described as a person of equal level of excellence and high similarity to the protagonist, target B as equal level of excellence and low similarity to the protagonist, and target C as low level of excellence and low similarity to the protagonist (target C). See Supplementary Table S1 for details.

### Questionnaire

We used the 15-item Passionate Love Scale (PLS; Hatfield and Sprecher, 1998) to measure the degree of love evoked by each participant in the vignettes. An example of an item in the PLS is, “I would rather be with him/her than anyone else…” Participants rated each item according to the degree of passionate love they perceived (1 = none; 9 = extremely passionate love). The PLS is suitable for individuals who are and are not in a relationship, and for individuals who have never been in a romantic relationship (Hatfield and Sprecher, 1986; Aron et al., 2005). The reliability and validity of this scale have been established in previous studies (Hatfield and Sprecher, 1986; Fehr, 1988; Hendrick and Hendrick, 1989; Fehr and Russell, 1991). Cronbach’s alpha coefficient was 0.94 in the present study.

### Procedures

#### Learning Materials

**FIGURE 1:**
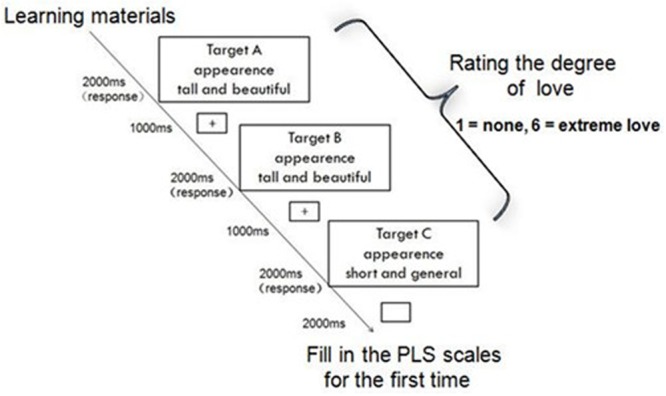
Part 1 consisted of three phases: studying the materials, rating on the computer, and completing the PLS. This figure presents a schematic depiction of the stimuli and rating task design of Part 1 (*love*). First, a fixation cross hair was presented for 1000 ms followed by the experimental stimuli (Lover A, Lover B, and Lover C) that were displayed for 2000 ms or until response. The top line in each stimuli-containing rectangle indicated a target person, the middle line indicated the domain of comparison (excellence and similarity), and the bottom line indicated the specific traits in these two domains.

**FIGURE 2:**
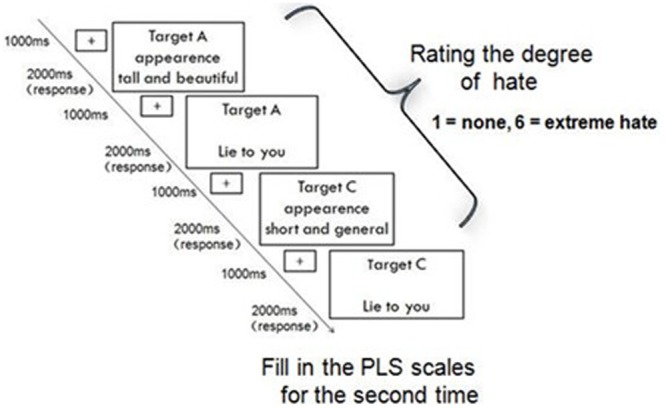
Part 2 consisted of two phases: rating on the computer and completing the PLS. This figure presents a schematic depiction of the stimuli and rating task design of Part 2 (*hate*). Specific traits of Lover A, Lover B, and Lover C were presented as in Part 1. Each trait was followed by a subsequent negative event, which was presented for 2000 ms or until response. The top line indicated a target person, and the bottom line indicated a negative event. A 1000 ms inter-stimulus interval was interleaved between each trait and negative event.

The experiment consisted of two parts. We induced feelings of love toward the targets in the participants (the protagonists) in Part 1 (**Figure 1**), and feelings of hate toward the targets in Part 2 (**Figure 2**).

First, participants were asked to read a story and imagine that they were the protagonist (see Supplementary Material). Next, the participants were asked to recall relevant key details about themselves by responding to sentences beginning with “I am…” Following this, participants were instructed to read three vignettes describing three different situations. Each vignette involved the protagonist and three targets. Participants were asked to recall the information relating to each target through free recall. Participants were then asked to imagine that they were in a romantic relationship with the target.

#### Ratings and Measurements

We used E-Prime 2.0 to present the items in a random order [we included 15 core items from each vignette into the reading materials of each target (see Supplementary Table S1)]. After the participants studied the materials, they completed the rating task on the computer and then completed the PLS in both Part 1 and Part 2. Participants gave one love score per item per target person in Part 1 and one hate score per negative event per target person in Part 2, as well as two PLS scores before and after the negative events.

In Part 1, we asked participants to imagine themselves as the protagonist when reading the scenario, and then rate each trait presented in terms of how much love they felt toward a target based on the presented features of the three targets (1 = none; 6 = extreme love). After that, we used the PLS to measure participants’ feelings of love with the three targets.

In Part 2 of the experiment, the background characteristics of A, B, and C were unchanged; however, we created vignettes in which the targets betrayed the protagonist, for example by having an affair with someone of the opposite sex (see the negative events in Supplementary Table S1). Participants were then asked to rate how much hate they felt toward A, B, and C (1 = none; 6 = extreme hate). Upon completion of Part 2, participants completed the PLS again to assess their feelings of love toward the three targets.

### Analysis

We used several analyses to test our hypotheses. The scores from love ratings, hate ratings, and the PLS items were averaged within subjects prior to the analyses. Specifically, we used one-way repeated measures of analysis of variance (ANOVA) to test for differences in participants’ love ratings, hate ratings, and PLS scores for targets A, B, and C; these analyses were conducted for scenarios with and without betrayal (Part 1 and 2). Simple effect tests were performed when the interaction effect was significant.

Additionally, we used a 3 (target: A, B, and C) × 2 (time: before vs. after) two-way repeated measures ANOVA to analyze the degree of love level perceived by the protagonist in relation to the three targets before and after the negative events. Next, we used a 3 (target: A, B, and C) × 2 (affect: love vs. hate) two-way repeated measures ANOVA to analyze the relationship between the love and hate scores. Tests of simple main effects were performed when an interaction effect was statistically significant. In addition, we used Pearson’s correlation analysis to test the correlations between scores for love and hate. Subsequently, we used partial correlations to examine the association between love and hate controlling for participants’ gender and age.

## Results

### Degree of Love

Across the different conditions (targets A, B, and C), the results of the one-way repeated measures ANOVA revealed significant differences in perceived feelings of love [*F*(2,116) = 985.710, *p* < 0.001, η^2^ = 0.944]. Further analyses of the simple main effects showed that the degree of love toward target A (5.53 ± 0.48) was significantly higher than that of target B (4.52 ± 0.54) [*F*(1,58) = 177.796, *p* < 0.001, η^2^ = 0.754], and the degree of love toward B was significantly higher than that of target C (1.66 ± 0.45) [*F*(1,58) = 977.526, *p* < 0.001, η^2^ = 0.944].

Additionally, across the different targets, the results of the one-way repeated measures ANOVA revealed significant differences in participants’ PLS scores of the three targets [*F*(2,116) = 450.352, *p* < 0.001, η^2^ = 0.886]. Further analyses of the simple main effects showed that the degree of passionate love toward target A (109.73 ± 11.80) was significantly higher than that of target B (93.46 ± 14.59) [*F*(1,58) = 60.263, *p* < 0.001, η^2^ = 0.510], and the degree of passionate love toward target B was significantly higher than that of target C (38.39 ± 20.40) [*F*(1,58) = 519.537, *p* < 0.001, η^2^ = 0.900].

### Degree of Hate

Across the different targets, the results of the one-way repeated measures ANOVA revealed significant differences in the degree of hate after the negative event manipulation [*F*(2,116) = 229.64, *p* < 0.001, η^2^ = 0.798]. Further analyses of the simple main effects showed that the degree of hate toward target A (5.25 ± 0.57) was significantly higher than that of target B (4.84 ± 0.55) [*F*(1,58) = 34.768, *p* < 0.001, η^2^ = 0.375], and the degree of hate toward target B was significantly higher than that of target C (3.02 ± 0.98) [*F*(1,58) = 216.921, *p* < 0.001, η^2^ = 0.789].

Across the different targets, the results of the one-way repeated measures ANOVA revealed significant differences of the overall PLS scores after the negative event manipulation [*F*(2,116) = 316.544, *p* < 0.001, η^2^ = 0.845]. Further analyses of the simple main effects showed that the PLS score for target A (88.95 ± 22.00) was significantly higher than that of target B (71.97 ± 21.83) [*F*(1,58) = 63.119, *p* < 0.001, η^2^ = 0.521], and the score for target B was significantly higher than that of target C (27.81 ± 14.39) [*F*(1,58) = 333.357, *p* < 0.001, η^2^ = 0.852].

The 3 (targets: A, B, C) × 2 (time: before vs. after) two-way repeated measures ANOVA revealed a significant target × time interaction [*F*(2,116) = 10.432, *p* < 0.001, η^2^ = 0.152] on PLS scores. Further simple main effect analyses revealed that after the negative event manipulation, participants’ love scores for target A was significantly lower than before the manipulation [A-Before: 109.73 ± 11.80, A-After: 88.95 ± 22.00; *F*(1,58) = 74.822, *p* < 0.001, η^2^ = 0.560]. Similarly, participants’ love scores for target B [B-Before: 93.46 ± 14.59, B-After: 71.97 ± 21.83; *F*(1,58) = 68.179, *p* < 0.001, η^2^ = 0.540] and target C were also significantly lower than before the manipulation [C-Before: 38.39 ± 20.40, C-After: 27.81 ± 14.39; *F*(1,58) = 27.842, *p* < 0.001, η^2^ = 0.324].

### Love and Hate

**FIGURE 3:**
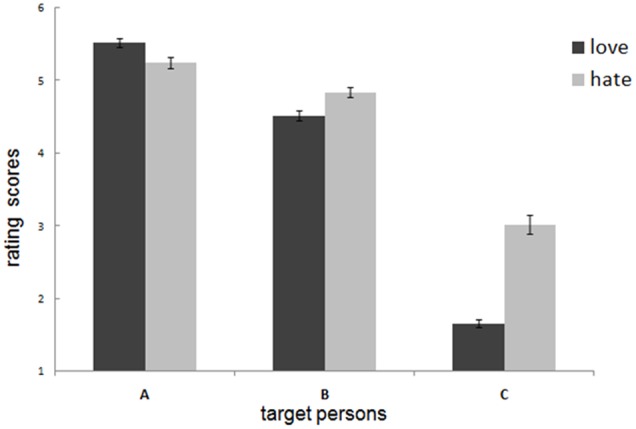
The love and hate level of all participants in response to the 3 (targets: A, B, C) × 2 (affect: love, hate) two-ways repeated measures ANOVA revealed a significant target × affect interaction. Error bars represent +1 standard error (SE). Participants’ degree of love for A (excellent and high similarity with the participants) was still higher than hate after negative events occurred, but the tendency for B (excellent and moderate similarity) and C (low excellence and low similarity) is opposite.

The 3 (targets: A, B, C) × 2 (affect: love vs. hate) two-way repeated measures ANOVA revealed a significant target × affect interaction [*F*(2,116) = 95.357, *p* < 0.001, η^2^ = 0.622]. Further simple effect analyses found that participants’ love of target A was significantly higher than that of hate, even if they were betrayed by target A [A-Love: 5.53 ± 0.48, A-Hate: 5.25 ± 0.57; *F*(1,58) = 17.889, *p* < 0.001, η^2^ = 0.236]. Conversely, participants’ love for target B was significantly lower than that of hate [B-Love: 4.52 ± 0.54, B-Hate: 4.84 ± 0.55; *F*(1,58) = 14.652, *p* < 0.001, η^2^ = 0.202]. Similarly, participants’ love for target C was also significantly lower than that of hate [C-Love: 1.66 ± 0.45, C-Hate: 3.02 ± 0.98; *F*(1,58) = 102.933, *p* < 0.001, η^2^ = 0.640] (**Figure 3**).

Furthermore, the Pearson correlation analyses showed significant relationships between participants’ love and hate toward target A (*r* = 0.55; *p* < 0.001). Participants’ love and hate toward target B (*r* = 0.29; *p* < 0.05). However, the correlation between participants’ love and hate toward target C was not significant (*r* = 0.12; *p* > 0.05). The corresponding partial correlation analyses revealed similar results (A: *r* = 0.48, *p* < 0.001; B: *r* = 0.27, *p* < 0.05; C: *r* = 0.12; *p* > 0.05).

## Discussion

This study used an experimental paradigm to study the relationship between romantic love and hate. The current study provided support for a link between the two affects and insights into the influence of similarity in romantic relationships. We found that people have different emotional reactions toward different target persons in the context of romantic love and hate. The relationship between romantic love and hate was revealed to be more complex than expected.

First, our results showed that feelings of love were influenced by similarity. That is, individuals, who were experimentally induced to experience feelings of love, felt stronger love toward someone of the opposite sex who was similar to them, thus, supporting our first hypothesis. Previous studies have examined whether similarity or complementarity played a more vital role in mutual attraction (Berscheid and Reis, 1998) and concluded that the former was more important. This view has also been supported by research looking at mate preferences (Luo and Klohnen, 2005) and quality of marital relationships (Hudson et al., 2014).

Previous studies had mostly recruited couples or partners who were already in a relationship, and there is little direct evidence on whether the similarity of the two individuals had a crucial role in the development of a romantic relationship. A recent study (Conroy-Beam et al., 2016) reported that mate value discrepancies predicted relationship satisfaction. To some extent, they considered the equivalence in social status between both partners to be an important factor relating to relationship satisfaction. In our study, however, when the participants were presented with two potential partners equal to them in excellence, participants perceived greater love for the one who was more similar to themselves. Relatedly, similarity also played an important role in mate selection. Our findings complemented the findings of other research in this area. Individuals who were similar to each other easily formed good impressions of each other within a short time. This finding combined with results of previous studies suggests that similarity plays a vital role in attraction, regardless of situations involving “love at first sight” or impressions based on long-term exchanges.

Second, we found significant associations between romantic love and hate in the context of a romantic relationship. When presented with negative events with three different target persons, participants most hated the person whom they had loved the most previously. Therefore, love and hate are indeed related. As Alford (2005) proposed, hate is an imitation of love and also a type of relationship with others and oneself. That is, in managing their relationships with others, people are at the same time managing themselves and their psyches (Alford, 2005). In the context of an individual’s love and hate, when the relationship one had developed with a particular partner was destroyed, the romantic love consequently turned into hate. Especially from the perspectives of young couples in romantic relationships, hate is also a reflection of love.

The relationship between love and hate can be explained from different perspectives. Romantic hate may be rooted in romantic jealousy. Previous research proposed emotional jealousy and cognitive jealousy as constituents of romantic jealousy. Emotional jealousy reflects the anger and fear of the individual in love, while cognitive jealousy mainly relates to the individual’s negative attitude to lovers (Yoshimura, 2004). Therefore, we speculate that it is a lover’s betrayal that causes anger and other negative emotions, resulting in hate. Moreover, cognitive jealousy is directly related to relationship dissatisfaction between lovers (Elphinston et al., 2013). Previous studies have also found a positive relationship between romantic love and jealousy. That is, the more one loves a person, the more sensitive one becomes when encountering threats to the relationship (Mathes and Severa, 1981; Orosz et al., 2015). Thus, individuals experience more love and more hatred toward the same lover.

The observed phenomenon of “the deeper the love, the deeper the hate” may also be attributed to the perception of equity imbalance. Researchers have proposed the concept of “perception of equity” based on equity theory and state that equity can be achieved by changing one’s perception of investments in the relationship or its results (Walster et al., 1973). According to equity theory, equity is calculated from both the individual’s inputs and the resulting outcomes (Hatfield et al., 1979). Thus, in our context, the more one loves a person, the more psychological investment one makes. However, when there is an imbalance between the individual’s inputs and outcomes, the perception of equity is lost, thus, resulting in a change of perception between hate and love.

At the same time, our results showed a significant interaction between targets (A vs. B vs. C) and affects (love vs. hate). Further analyses revealed that an individual’s degree of love for target A (equal excellence and high similarity with the protagonist) is still higher than the degree of hate after negative event manipulation, but the results were reversed for target B (equal excellence and low similarity with the protagonist) and target C (unequal excellence and low similarity with the protagonist). In other words, although the three targets were associated with the same negative events, the level of hatred varied across the three targets. If, initially, the individual loved the target the most, the degree of love is still higher than that of hate after the negative event. However, when the individual did not love the target as much initially, the degree of love would be markedly lower than that of hate.

These results illustrate the complexity associated with romantic love and hate. People have different emotional reactions toward different target persons in the context of romantic love and hate. For the person whom one loves the most or even hates, love may still be dominant in the context of betrayal. This hate is a reflection of love and a feeling of sorrow. However, for the person one does not love, feelings of hate are stronger than those of love. This hate perhaps has its roots in the moral dimension, which mainly concern social judgments about the quality of a person. This is why people experience such pain upon betrayal in a romantic relationship.

Graham and Clark (2006) found that individuals who look at a relationship as “all good” or “all bad” have lower self-esteem compared to others. These individuals also have long-term concerns about whether their partners are willing to accept them in a closed relationship. The authors proffered this as the reason behind love and hate, and that this phenomenon could be observed in any relationship. Needless to say, the complex precursors of love and hate can be interpreted in many ways. Perhaps as some of the most ubiquitous emotions, people need to comprehend and explain love and hate objectively and rationally. Although we study the nature of love and hate from a rational point of view and from an emotional perspective to explain the precursors of these two basic emotions, humans are emotional beings.

In summary, we need to comprehend the relationship between love and hate both rationally and emotionally. If we pay close attention to hate, we can better understand love (Tjeltveit, 2003). This idea justified us carrying out the current study. However, there are three limitations to this study. First, even though we emphasized that the protagonist would be described in three different relationships in different periods of life, this manipulation could not guarantee that participants could generate independent feelings of love for the three target persons. Second, in order to maximize external validity of the study, we did not control for participants’ current relationship status. In our future research, we may explore whether relationship status predicts feelings of love and hate using this experimental paradigm. Third, the findings of the current study were also limited by the manipulation of similarity between the participants and the three targets. The use of vignettes meant that the manipulation of similarity might have partly depended on how well the participants were able to imagine themselves as the protagonist in the vignettes.

## Conclusion

Our results supported the idea that “the deeper the love, the deeper the hate,” and suggested similarity as a crucial factor influencing feelings of love and hate. In addition, people have different emotional reactions toward different people in the context of romantic love and hate. For the person whom one loves or hates the most, love may still be dominant in the context of betrayal. However, for the person one does not love, feelings of hatred are stronger than those of love. This study also provided support for the relationship between romantic love and hate, and highlighted the important role of similarity in moderating the relationship between love and hate.

## Ethics Statement

The present study was approved by the Ethic Committee of the School of Psychology at South China Normal University. Each participant volunteered to take part in this study and provided written informed consent before the start of the experiment.

## Author Contributions

WJ: study design, data collection, data analysis, and paper writing. YX and ML: study design and paper writing.

## Conflict of Interest Statement

The authors declare that the research was conducted in the absence of any commercial or financial relationships that could be construed as a potential conflict of interest.
