# Supplementary material for: The Deeper the Love, the Deeper the Hate
Source: Front Psychol. 2017 Dec 7;8:1940. doi: 10.3389/fpsyg.2017.01940 (PMC5725944; doi:10.3389/fpsyg.2017.01940)
Supplement: Supplementary file 1 [file Presentation_1.pdf]

Supplementary Material

The Deeper the Love, the Deeper the Hate

Jin-Wang, Yan-hui Xiang*, Lei Mo*

*** Correspondence:** Corresponding Authors: molei@m.scnu.edu.cn, xyh914@163.com

Supplementary Appendix

The background characteristics of the protagonist and three targets of A, B and C.

Vignettes for male participants

Female participants read the same vignettes with slight modifications (within the parentheses) in the appearance, personality traits, and accomplishment

Reading Materials

The protagonist

I am a student of this university. I am hoping to get into a romantic relationship. I have a handsome (pretty) face. I have a tall and slim figure. I have my own life goals to pursue, and I think success depends on my own effort. I have an excellent academic record, and I have been elected to be the student union chairman. Through my hard work, I hope to get a job that I desire upon graduation. I will continue to work hard in the future, so as to own a sea-view apartment, and have a home that belongs to me. Apart from my studies, I like to travel around the world; travelling brings me a lot of new experience and fun. I am particularly good at playing the cello (piano). I am a musically cultured person who has a unique and humble style. I have sharp wits (am gentle and kind), like to make friends with like-minded people, teachers and classmates like me a lot.I hope to receive the blessings and love from both parties' parents. In love, I hope that both of us can be integrated into each other's family. I pay great attention to details. When I am sick, I expect meticulous care and companion from my partner. I hope to receive a

specially prepared gift from my partner on my birthday. The gift need not be expensive as long as it is sincere. Every single gift carries the memories that we have been through together. I hope to meet this person I have in mind on the university campus, and start a beautiful romantic relationship.

Target A: (High similarity, High excellence)

She has a pretty face, a tall and slim figure. She has goals to pursue in her life and thinks success depends on her own effort. She has an excellent academic record and has been elected to be the student union chairman. She loves this bustling and beautiful city. She hopes to continuously work hard, so as to be able to own a sea-view apartment. She is rather disciplined during her spare time; she likes to work out. Apart from her studies, she likes to travel, and often goes for trips on the weekends or during the summer holidays. She is particularly good at playing the piano and has a unique yet humble style. She is gentle and kind, optimistic and open-minded. She likes to make friends with like-minded people, teachers and classmates like her very much. She hopes to receive the blessings and love from both parties' parents, and also hopes that her partner will be able to obtain the trust and love from her parents. In love, she pays great attention to the details. To her, having meticulous care and companionship in times of illness, and receiving a specially prepared gift from her partner are small things that will give her a wonderful feeling of love.

Target B: (Low similarity, High excellence)

She has a pretty face, a tall and slim figure. She believes that one needs to go with the flow in order to be successful. Her academic results are average. She stood for class monitor elections previously but was not elected. She hopes to be able to buy village garden home some day, and lead a comfortable life. She is not disciplined during her spare time; she does not like to work out. Apart from her studies, she likes to indulge herself in food and drinks together with her friends. During the weekends and summer holidays, she hangs out with the same group of friends. She likes to play the oboe and has a few favorites' pieces that she can play. She has an adventurous personality; she is a social and optimistic person. She likes to make friends and has relatively good relationships with others. In love, she feels that parents' expectations are not important. She feels that when she is in love, she is not bothered by any details, sometimes she will forget her partner's birthday.

Target C: (Low similarity, Low excellence)

She has an average look; she is slightly plump and is not tall. She believes that success depends on having good contacts, and one's hard work is of little significance. Her academic results are average. Although she has taken part in a class cadre elections, she was not elected. She feels fine as long as she has a place to

live in, in the future. In life, she just muddles along.She often stays up all night and does not like to work out. Apart from her studies, she does not have any special hobbies. She often stays at home on the weekends or during the holidays. She is self-centered, taciturn, does not have many friends and has average interpersonal relations. In love, she does not care much about how her parents think about her future mate, as long as she loves him. She is not too bothered by details in a relationship and does not remember her partner's birthday.

Measures

Supplementary Table S1

Experiment stimuli list

	Comparative dimension	Target A high similarity, high excellence	Target B low similarity, high excellence	Target C low similarity, low excellence	Negative events
1	Appearance (male)	Tall and handsome	Tall and handsome	Short and general	Often chats with the opposite sex secretly
1	Appearance (female)	Tall and beautiful	Tall and beautiful	Short and general	Contacts ex-boyfriend in private.
2	Values	Success depends on one's effort	Success depends on going with the flow	Success depends on having good contacts	Contacts ex- girlfriend in private.
3	Housing target	Sea view apartment	Village garden	Any place to stay in	Doesn't admit that she has a boyfriend.
4	Interest	Travelling	Indoor activities	Stays at home	Doesn't admit that he has a girlfriend.

5	Personal ability	A student leader	Intern of big company	Failed to be elected as a class chairman	Takes you as the replacement of her former boyfriend.
6	Personality traits (male)	Careful and considerate	Serious and pragmatic	Independent	Takes you as the replacement of his former girlfriend
6	Personality traits (female)	Gentle and kind	Serious and pragmatic	Independent	Uses dating websites secretly
7	Accomplishment (male)	Plays the cello	Wons calligraphy competitions many times	None	After your wealth
7	Accomplishment (female)	Plays the piano	Wons dance competitions many times	None	Often lies to you.
8	Social relations	Many friends in academic circles	Many friends in business circles	Takes little initiative to make friends	Does not keep his (her) word
9	Parent's opinion	Very important	For reference only	Not important	Does not like your parents.
10	Romance	Carefully prepared birthday gift	Birthday surprises	Often forgets your birthday	Secretly goes out on dates with the opposite sex
11	Personality traits	Outgoing and active	Mature and introverted	Taciturn	Secretly fancies one of your room mates.
12	Health awareness	Works out at gyms	Often go hiking	Does not like to exercise	Agrees to be on a blind date as arranged by his (her) family
13	Love attitudes	Dedicated love	Love depends on fate	Does not believe in love	Does not for seeing a future with you upon graduation
14	Career orientation	Professor	Business manager	Teacher in a primary or	Thinks you are too old fashioned

				secondary school	
15	Lifestyle	Modern	Traditional	Has no special hobbies	Does not want to marry you
